# Asynchronous transcriptional silencing of individual retroviral genomes in embryonic cells

**DOI:** 10.1186/1742-4690-11-31

**Published:** 2014-04-17

**Authors:** Sharon Schlesinger, Eran Meshorer, Stephen P Goff

**Affiliations:** 1 Department of Biochemistry and Molecular Biophysics, Columbia University, HHSC 1310C, 701 W. 168th St., New York, NY 10032, USA; 2 Department of Microbiology and Immunology, Columbia University, HHSC 1310C, 701 W. 168th St., New York, NY 10032, USA; 3 Howard Hughes Medical Institute, Columbia University, HHSC 1310C, 701 W. 168th St., New York, NY 10032, USA; 4 Department of Genetics, The Institute of Life Sciences, The Hebrew University of Jerusalem, Edmond J. Safra (Givat Ram) Campus, Jerusalem 91904, Israel

**Keywords:** Embryonic stem cells, Transcriptional silencing, Chromatin modifications

## Abstract

**Background:**

Retroviral DNAs are profoundly silenced at the transcriptional level in embryonic cell types. The transcriptional profile of pluripotent stem cells has been demonstrated to be extremely heterogeneous from cell to cell, and how the silencing of retroviral DNAs is achieved is not yet well characterized.

**Results:**

In the current study, we investigated the transcriptional silencing dynamics in stem cells by independently monitoring the expression of two Moloney murine leukemia virus (MMLV) retroviral vectors newly introduced into embryonic carcinoma (EC) cells. Although MMLV is efficiently silenced by epigenetic mechanisms in most such cells, a small number of the doubly-transduced EC cells transiently show double-positive proviral expression. These cells were sorted and their expression patterns were studied over time as silencing is established.

**Conclusions:**

Our data suggest that retroviral silencing occurs stochastically, in an individual locus-specific fashion, and often without synchronous silencing of both viruses in the same cells. Surprisingly, the chromatin modifications that mark the silenced proviruses are unchanged even in cells that temporarily escape silencing. This local silencing effect is a feature of stem cell epigenomic regulation that has not previously been revealed.

## Background

Developmental programs are executed by the tightly controlled and temporally coordinated transcriptional regulation of large sets of genes. As cells move through developmental stages during embryogenesis, groups of genes are often synchronously turned on and off to induce specific changes in cell phenotype and in the capabilities needed for tissue and organ formation. This gene regulation is mediated by trans-acting transcription factors, and is accompanied by long-lasting alterations in chromatin folding, histone modifications, and DNA methylation at the genomic regions of the genes being regulated. While the sets of coordinately regulated genes may sometimes be genetically linked at a single chromosomal region, they are most often unlinked and dispersed at many disparate locations on many chromosomes. In these cases the trans-acting machinery must find and act on the multiple loci in a coordinated, synchronous manner. The timing of these regulatory events can be tightly synchronous, but there is some inherent noise or variability in the regulatory machinery, and thus there can be considerable cell-to-cell fluctuation in gene expression [1]. As a result, even genetically identical cells in a largely homogeneous environment can display different phenotypes. This variability can arise either from stochastic fluctuations in biochemical reactions that regulate gene expression in “trans” (“extrinsic” variability), or from preexisting epigenetic heterogeneity of the genes being regulated (“intrinsic” variability) [2]. These fluctuations in gene expression can play useful and important roles in development. Fate choice in pluripotent stem cells involves the modulation of networks of transcription factors occurring in an apparently stochastic fashion and resulting in a heterogeneous cell population [1,3,4]. The heterogeneity in expression of many components of the transcriptional network was shown to be a key feature of pluripotency [5-7].

Retroviral DNAs provide a unique tool for the analysis of gene expression because they can be introduced into the genome at will by infection, and because they are introduced as “naked”, unmodified, newly synthesized DNA that is assembled into chromatin by the addition of new nucleosomes. Vector genomes that express reporter genes are powerful tools to monitor regulation of transcription in infected cells [8]. Regulatory elements within the newly integrated DNA have to be recognized by trans-acting factors, and the subsequent expression pattern of the DNA has to be established by those factors and by the preexisting state of the chromatin around the site of insertions. Although retroviral expression can be affected by the site of integration, it is most profoundly influenced by the cell type. In differentiated cells, infection by the MLVs typically results in high-level constitutive expression of the provirus, while in mouse embryonic stem cells, embryonic carcinoma cell lines, and other primitive cell types, the viral DNA is heavily silenced at the transcriptional level [9-11]. The silencing of retrovirus DNAs occurs rapidly when the virus contains a conserved sequence element termed the proline primer binding site (PBS), an 18-nucleotide sequence complementary to the 3′ end of proline tRNA, the tRNA primer used for initiation of reverse transcription by MMLV [12,13]. Silencing at the proline PBS is mediated by the zinc finger DNA binding protein ZFP809, and by a well-characterized silencing protein, Trim28/Kap-1/Tif1b, which interacts with ZFP809 [14,15]. Retroviral vectors utilizing alternative PBS sequences that are not recognized by this silencing machinery escape the rapid silencing but are still subject to some transcriptional repression [16,17]. The machinery mediating this silencing is also used to repress or regulate expression of many of the endogenous proviruses resident in the mouse genome, most strikingly the so-called IAP elements [18], through the DNA binding factor YY1 [19]. We note, however, that not all endogenous proviruses are equally or even similarly regulated in this pattern, and that some retroelements show the inverse behavior, expressing well in ES cells and not in differentiated cells; these include the HERV-H proviruses in human cells [20] and the virus-related L1td1/Ecat11 gene in mouse [21]. For these elements other regulatory factors must be in play.

**Figure 1:**
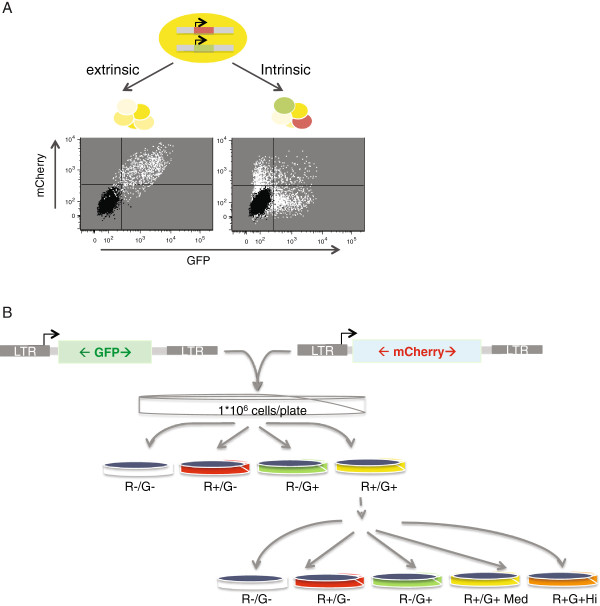
**Proviral silencing in embryonic cells – hypothesis and experimental procedure. (A)** Scheme illustrating two alternatives for the proviral escape from silencing: if the regulation of expression is fully “extrinsic”, both proviruses integrated in one cell will be expressed or silenced in a synchronous and coordinated manner. That pattern might, for example, be indicative of a distinctive cellular state of the transcriptional network. A hypothetical dot plot describing this type of population is illustrated on the left. An alternative is an intrinsic or gene-specific control of the expression. Here the two proviruses in one cell will each be controlled independently and thus can be expressed asynchronously, independently of each other. This will lead to a heterogeneous population of cells expressing either red, green, both, or no marker gene, as illustrated in the dot plot on the right. **(B)** Illustration of the experimental procedure: one MMLV viral vector carrying a GFP reporter and a second one carrying an mCherry reporter were each packaged into virus particles and used together to infect F9 EC cells at high MOI. The infected cells were grown for 6 days and then sorted by fluorescence-activated cell sorting (FACS) to four different populations. The populations were then grown in culture, and most centrally for this study, after three weeks the initially double positive population was sorted a second time, this time into five different populations. These cells were used in the experiments described herein.

In previous studies we showed that although most ES cells silence the incoming retroviral DNA, a small subpopulation of infected cells (‘escapees’) evade silencing [19]. When sorted and re-cultured, this rare population of cells rapidly silences the provirus, and within 3 days exhibits the expression pattern of the original population. Here, to determine whether the rate-determining parameters of silencing are imposed globally (and thus synchronously) or only locally (and thus asynchronously) we made use of a two-virus/two-color MMLV infection system, in which cells were infected with two MMLV vectors carrying GFP and mCherry reporter genes, and we examined the pattern of expression of each gene over time on a cell-by-cell basis. Our results suggest that proviral genes are in this setting silenced independently and stochastically, and not in a global, genome-wide silencing mechanism (Figure 1A).

## Results

### F9 cells infected with two reporter viruses rapidly and efficiently silence both proviruses

**Figure 2:**
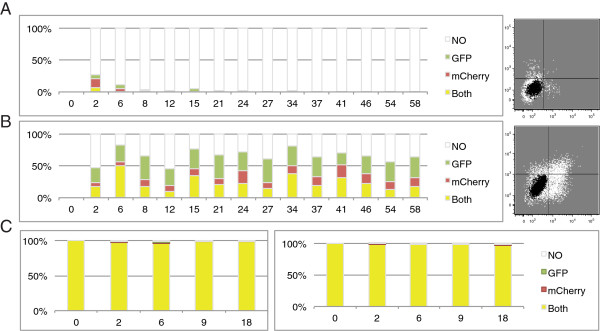
**Kinetics of silencing. (A-C)** Flow analysis of the infected cells in the days after infection. The stacked bar graph reports the% of cell population expressing both viruses (yellow), the mCherry alone (red), the GFP alone (green) or none (white) on the y-axis. X-axis represents days after infection. **(A)** F9 EC cells infected with PBSpro – GFP and mCherry viruses or **(B)** PBSgln – GFP and mCherry viruses. **(C)** NIH3T3 cells infected with the same PBSpro (left panel) or PBSgln (right panel) virus concentration that was used to infect the F9 cells (see also Additional file 1: Figure S1).

To monitor the stability of retroviral restriction in embryonic cells, two MMLV viral vectors, one containing a GFP gene and one an mCherry reporter gene, were separately packaged into virus particles, and the viruses were then mixed and used to co-infect F9 embryonic carcinoma (EC) cells at a multiplicity of infection (MOI) of approximately 2. Cells of the F9 line, originally isolated from a murine teratocarcinoma [22,23], are pluripotent but not totipotent, and are often used as a surrogate for authentic embryonic stem cells. The moderately high MOI ensured that the majority of the cells were infected with both viruses, and analysis of the DNA copy number confirmed that most cells received both proviruses (Additional file 1: Figure S1). Cells were analyzed by flow cytometry at various times after infection to determine the percent of double positive (R + G+), single mCherry positive (R + G-), single GFP positive (R-G+) and double negative (R-G-) cells (see Figure 1B for experimental design). The experiment was carried out with pairs of virus vectors utilizing either the wild-type PBSpro sequence or a PBSgln. The PBSpro viruses were profoundly and rapidly silenced in F9 EC cells (Figure 2A). The PBSgln viruses were also partially silenced in these cells, typically giving rise to 30-50% mCherry or GFP-positive cells (Figure 2B), as previously observed. In contrast, differentiated cells - virus-susceptible NIH3T3 fibroblasts – infected at the same multiplicity showed efficient infection and stable expression of both viral genomes (Figure 2C).

### Transiently-expressing PBSpro viruses are rapidly silenced, but not globally and synchronously

**Figure 3:**
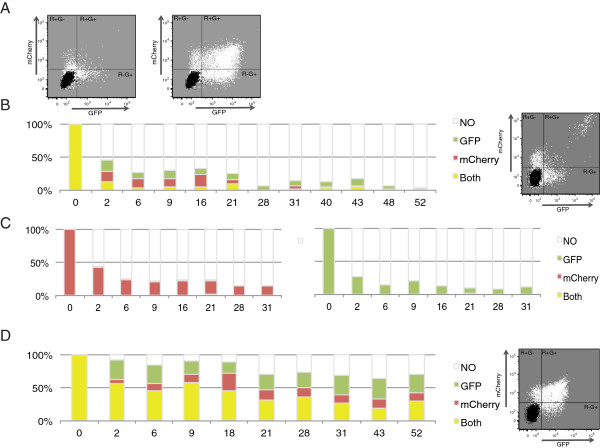
**Following an initial sort, silencing is re-established in a stochastic, unsynchronized manner. (A)** Flow analysis dot plot of the PBSpro viruses (on the left) and PBSgln viruses (on the right) on day 6 after infection (white dots) and un-infected control (black dots). **(B)** Flow analysis of the double positive sorted cells for viral expression spanning 52 days after sorting. On the right – an example of the flow analysis dot plot as recorded 21 days after sorting. **(C)** Flow analysis of the PBSpro mCherry (left) or GFP positive cells (right) spanning 31 days after sorting. **(D)** Flow analysis of the PBSgln infected F9 cells, double positive sorted population. On the right – an example of the flow analysis dot plot as recorded 21 days after sorting.

Fluctuations in retroviral silencing have been reported before for endogenous [24] and exogenous [25] retroviruses. In order to characterize the nature of the phenomenon, we sorted F9 cells infected with mCherry and GFP retroviral vectors by fluorescence-activated cell sorting (FACS). Cells were sorted 6 days after infection, and four isolated cell populations were cultured and analyzed for continued mCherry and GFP expression by cytometry (Figure 3A). F9 cells sorted for initial expression of both mCherry and GFP PBSpro (R + G+) viruses rapidly and efficiently silenced the expression of each virus (Figure 3B) in a manner similar to the individual silencing of each virus alone (Figure 3C). The silencing of the two viruses in a single cell did not occur in a synchronized, coordinated manner, but rather occurred in an asynchronous, stochastic manner. Thus, during the course of silencing, cells often passed through a single-positive GFP or mCherry state before ultimately reaching a double-negative state. F9 cells sorted for double-positive expression of two PBSgln viruses only partially silenced their expression, with ~70% of cells remaining GFP-positive after 15 passages, over a span of 50 days in culture (Figure 3D). The PBSgln infected cells were able to independently shut down expression of one or the other of the two viruses, as the wt PBS infected cells do. We subcloned the double-positive sorted cells arising after infection with PBSpro virus and followed the expression profiles of several independent clonal populations by flow cytometry (examples of clones shown in Additional file 2: Figure S2A-E). The clones showed major variation in the rate and extent of silencing, perhaps attributable to the different sites of integration of the proviruses in each clone. Nevertheless, the majority of the cells in all the clones eventually totally silenced both viruses. As seen in the general population, the double-positive cells in the various clones often progressed through a single-positive state en route to the double-negative state. Differentiated NIH3T3 cells infected with pairs of either PBSpro or PBSgln viruses and sorted as double positives, remained fully positive for >15 passages (data not shown).

### Isolation of two double-positive populations – one stochastically silencing and one permanently expressing

**Figure 4:**
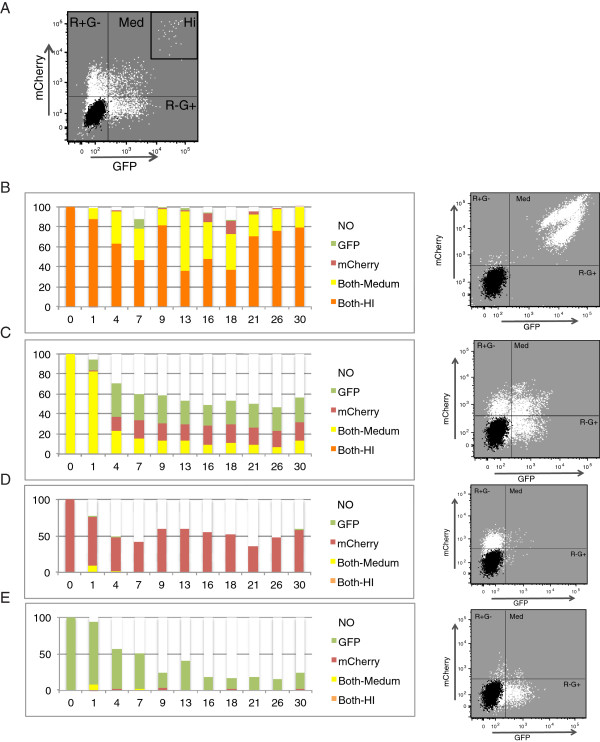
**After a second sort, a subpopulation of stable expressing cells can be isolated and characterized. (A)** Flow analysis of cells infected with PBSpro virus, first sorted as double positive, and then monitored on the day of the second sorting. Infected (white) and uninfected control cells (black), and gate names, are indicated. **(B-D)** Flow analysis for 30 days after second FACS sorting of cells from **(B)** High expressing- double positive cells. **(C)** Medium expressing- double positive cells after second sorting. **(D)** mCherry-only positive cell and **(E)** GFP only positive cells. On the right – an example of the flow analysis dot plot as recorded 26 days after second sorting.

In an attempt to isolate a minor population of cells that displayed a global and stable escape from silencing, we sorted the initial double positive population from infection with PBSpro virus a second time, 22 days after the initial sort (Figure 4A). This time, we sorted out five populations that were again cultured and analyzed over time for continued mCherry and GFP expression by flow cytometry (Figure 4B-E). Cells sorted as double-negative remained negative. Cells sorted as single-positive silenced the reporter gene over time (Figure 4D,E). Cells sorted for moderate levels of expression of both mCherry and GFP PBSpro (R + G + Med) viruses again silenced their proviral reporter expressions (Figure 4C) in a manner similar to the individual silencing of each virus alone, and similar to the silencing of the initially sorted double-positive cells. In contrast, rare cells sorted for very high level expression of mCherry and GFP (Hi) displayed a stable expression over time, with almost no silencing (Figure 4B). This small and rare cell population, assuming a normal rate of cell growth during their expansion in culture, would have originated from less than 100 cells of the original infected population. Thus, these cells may represent a pool with special proviral integration sites or DNA copy number. Indeed, analysis of the viral DNA copy number showed that these cells on average carried approximately 10 times more viral copies than the other cell populations (Additional file 1: Figure S1A). This high copy number itself, or the higher probability of a provirus integrating at rare chromosomal regions, may be responsible at least in part for the stable escape from silencing. However, examination of the endogenous proviral DNA expression in these cells (see below) suggests that overall retroviral silencing *per se* may have been lost in these cells.

### Stochastically expressed proviruses are still marked by repressive epigenetic marks

**Figure 5:**
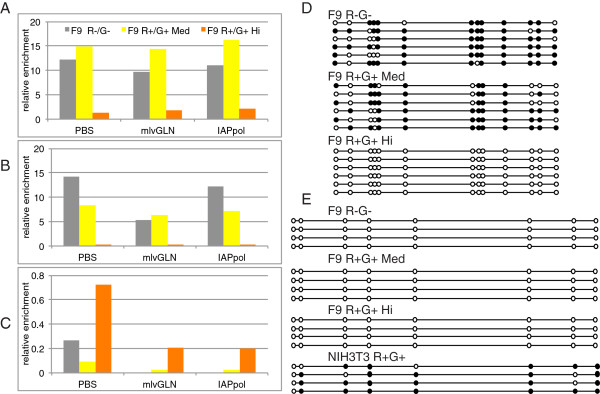
**Chromatin and DNA methylation assessment of the High, Medium, and Negative expressing sorted cells. (A)** ChIP–based measurement of H3K9me3, **(B)** H3K27me3 and **(C)** H3K4me3 at the viral U5-PBS region, ERV class I (mlvGLN) and ERV class II (IAPpol) in sorted populations. Enrichment values are relative to total input and normalized to the signal of negative control (Gapdh or Necap1). **(D)** Bisulfite sequencing analysis of the 5′LTR of the infecting virus was conducted on genomic DNA isolated from the sorted cells. Open and filled circles represent unmethylated or methylated cytosines, respectively. **(E)** Bisulfite sequencing analysis of Oct4 was used as control.

To determine the chromatin state of the active loci, we analyzed the genomic regions of the five twice-sorted cell populations by chromatin immunoprecipitation (ChIP) for active (H3K4me4) and suppressive (H3K9me3; H3K27me3) histone modifications. In F9 cells sorted as double positive in the first sort and double negative in the second sort, the proviral sequences were highly enriched for H3K9me3 (Figure 5A) and H3K27me3 (Figure 5B) marks, indicative of a closed or repressed chromatin conformation, as in unsorted, actively silencing F9 populations [19]. Surprisingly, the moderately double positive expressing cell population (R + G + Med) also displayed suppressed chromatin marks, suggesting that although transiently positive, these cells had already marked the majority of the proviruses for subsequent silencing. Thus, basal and stochastic expression of the reporter genes can occur while the majority of the proviruses are packaged in a closed chromatin conformation. In contrast to the partially or fully silenced clones, the subpopulation of very high expressers (R + G + Hi) maintained an open chromatin conformation of the provirus, with no H3K9me3 and H3K27me3 marks and high levels of H3K4me3 (Figure 5C). This was true not only for the infecting MMLV proviruses, but also for ERVs from class I and II sequences, represented by the MLVgln ERVs and the IAPs, respectively (Figure 5C). Thus, in these stable high-expressing escapees, both exogenous and endogenous proviruses are maintained in an open chromatin structure. To further characterize the epigenetic status of this high-expressing double positive population, we analyzed the DNA methylation states of the U5-LTR region in the different sorted populations (Figure 5D). The percentage of methylated CpGs was comparable between the double-negative and the moderately-expressing double positive populations (77% versus 68%) but was completely absent in the high-expressing double-positive population (0%; Figure 5D). The levels of CpG methylation in the single- and medium double-positive cell populations were stable over time and similar in individual subclones (Additional file 3: Figure S3). CpG methylation of the pluripotency gene Oct4 was low in the high-expressing population as in normal embryonic cells, demonstrating that the changes in the methylation status are specific to the proviruses and that these cells are not differentiated (Figure 5E). Analysis of the expression profiles of these cells by RT-PCR showed that the pluripotency markers Oct4 and Nanog were still expressed, confirming that the cells were not differentiating (data not shown). We conclude that the stochastic and temporary escape from silencing is transiently overriding the closed chromatin conformation of the genomic region containing the majority of the proviruses, while the more stable and persistently expressing cells have lost their epigenetic silencing modifications of both exogenous and endogenous proviruses such that these viral DNAs are marked by active chromatin and DNA modifications.

### Different histone modifications on the proviral alleles

**Figure 6:**
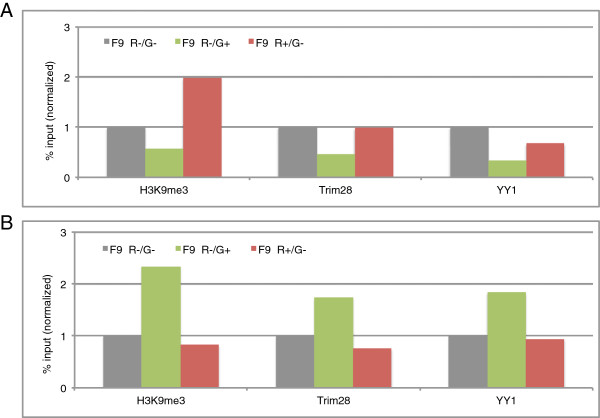
**The two proviral copies are independently and differentially bound by the silencing complex. (A)** ChIP–based measurement of H3K9me3 mark, Trim28 and YY1 present on the proviral DNAs in sorted populations. ChIP data is determined as recovered DNA relative to input DNA, and the ratio between occupancy on the GFP gene to the occupancy on the average of both proviruses in the U5-PBS region is presented. The values are finally presented as compared with the values for the double-negative sorted population, with the double-negative cells normalized to 1. **(B)** Same as A, ratio between occupancy on mCherry to that on the total U5-PBS region is presented, again with the ratio for the double-negative sorted cells normalized to 1.

To determine whether the stochastic changes in viral expression were correlated with changes in the chromatin state, the one-color expressing cell populations were analyzed by chromatin immunoprecipitation (ChIP). As these cells were sorted as double-positive in the first sort, each cell contains at least one of each provirus, which was at least transiently expressed. After 22 days in culture, many of these cells had silenced one proviral copy but not the other one. Expression of the positively-sorted allele was not stable and was silenced after several weeks (Figure 4D and E). We used ChIP assays to determine the levels of H3K9me3 and the occupancy of the silencing factors Trim28 and YY1 on the GFP or mCherry vector only (using specific primers) and on the LTR region of both viral vectors (using common, shared, U5-PBS primers). The results are presented as the ratio between the enrichment on the GFP gene only to that on the average of both copies (GFP/U5-PBS; Figure 6A), or the ratio between the enrichment on the mCherry gene only to that on the average of both copies (mCherry/U5-PBS; Figure 6B). We compared this ratio for the two single-positive sorted cell populations to the ratio in the double-negative cells (normalized at 1). The GFP-positive-only population displayed an approximately two-fold less enrichment of the silencing factors on the active GFP allele relative to the enrichment on the averaged LTRs, as compared to the double-negative cells (Figure 6A). The enrichment on the silent mCherry locus in these GFP-positive only cells was higher or similar to that in the double-negative controls. The mCherry only-expressing cells display the opposite pattern: the silencing marks and factors were slightly lower on the active Cherry gene relative to the averaged LTRs, and much higher on the silent GFP gene. Thus, the transiently active alleles showed somewhat lower occupancy of the silencing factors than the average proviruses in the same cell populations. It is possible that although in general most of the proviruses are packaged into heterochromatin and occupied by silencing factors most of the time, the double infection and sorting method enable us to observe the slightly “open window” that allows the fluctuation phenotype of transient expression to occur.

## Discussion

The experiments above show that embryonic cell populations that have silenced most proviruses still contain some cells that transiently transcribe the viral DNAs at low frequencies, and then quickly shut them off. We can recover such expressing cells by sorting, and we can watch the kinetics of the shutoff on a cell-by-cell basis. The results suggest that the silencing of retroviruses in these cells is most often a stochastic event mediated through local changes occurring independently at each provirus, and is not mediated by a global change in the state of the cell that acts simultaneously in a coordinated way on all the proviruses in a cell. This characteristic seems to be true whether the cells utilize the highly efficient PBSpro-directed machinery, or the less efficient PBS-independent machinery. The local, *cis*-acting nature of the suppression seen in these cells is reminiscent of the *cis*-acting control of expression seen in the course of silencing of MLV vectors introduced in successive infections [26,27].

EC cells have long been known for their ability to silence incoming viral DNAs, first documented for the silencing of the papova viruses [28]. This behavior is manifested by many mouse EC lines, including the F9 studied here and the PCC4 line [14], as well as authentic embryonic stem cell populations. The silencing of retroviral DNAs in our EC cells is moderately rapid, with expression decreasing sharply over the course of a few days at most. We note that the true time of transcriptional shutoff may be even faster than the apparent time course, because the GFP and mCherry proteins are relatively stable and the observed loss of fluorescent signal cannot be faster than the rate of decay of the accumulated proteins, likely occurring over several hours. Similarly, the appearance of a single-positive cell from a previously double-positive cell requires that the difference in time between the shutoff of the two reporters must be at least several hours. Thus, the asynchrony in the shutoff that we see in our experiments is very significant.

It was previously shown that genes expressed at low levels tend to be bound by fewer transcriptional regulators, both activators and repressors, which results in high cell-to-cell variability [29]. This variability has been proposed to be the result of the transcriptional regulators fluctuations [30], suggesting that as the number of different regulators increases, target sensitivity and variability of expression decreases [31]. In our experiments, the variability in timing of the establishment of silencing does not strongly depend on the level of expression. Both the efficiently silenced PBSpro and the less efficiently silenced PBSgln viruses are stochastically silenced. Thus, both the potent silencing mediated by the PBSpro-dependent ZFP809/TRIM28 system, and the silencing of the less potent non-PBS system, show similar asynchronous silencing. The behavior of both these systems may result from local fluctuations in the regulators acting at each provirus.

The status of the histone modifications and DNA methylation at the proviruses suggests that our EC cells typically mark the incoming viral DNAs for silencing soon after infection. Even those cells selected as transiently double-positive and single-positive, as well as double-negative, contain proviral DNAs exhibiting the chromatin and DNA marks of silent genes. Thus, the brief expression of the proviruses in these cells occurs in spite of these marks, and relatively quickly responds to the marks by shutting down. It is also possible that because most cells contain more than one copy of each virus, and because only one copy in the double-positive population may be expressing at any given moment, the silencing chromatin marks that we observe are present on the silent alleles. The expressed alleles thus may be at least partially depleted of the silencing marks, and this loss may be obscured by the silent alleles.

The rare population of cells that are stable expressors of both GFP and mCherry, recovered as high expressers after two sorts separated by many days in culture, are unusual escapees of the silencing process. These cells may have been selected to have proviral integrations at special loci. They may be similar to an EC line described by the Jaenisch lab, isolated after selection for expression of a marker gene delivered by a vector with a proline PBS [32]. It is noteworthy that the proviruses in these cells show the histone marks, and DNA methylation status, of completely open chromatin and highly expressed genes. But the fact that these cells also show altered chromatin marks and DNA methylation status for their endogenous retrovirus DNAs strongly suggests that they have undergone a global change in their silencing machinery acting in *trans*, and that the expression of both GFP and mCherry vectors is not solely a consequence of their integration sites. We surmise that the cell sorting regimen has selected for a cell population that has stably lost some components of the silencing system. Further analysis of the repertoire of expressed genes in these cells may ultimately allow for identification of the basis of their permissivity.

## Conclusions

The silencing of newly introduced retroviral genomes in embryonic cells is achieved by the recognition of the viral DNAs by transcription factors and chromatin modifying machinery. This machinery acts on the independently integrated proviruses in a temporally asynchronous manner, such that each provirus can be silenced without strict coordination with another provirus in the same cell. The findings suggest that there is a stochastic aspect to the determination of the chromatin modifications that are imposed on each provirus. Rare clones in which more profound escape from silencing arise by more dramatic global changes in the embryonic cell state.

## Methods

### Cells and viruses

F9 and NIH3T3 cells were cultured in DMEM with 10% FBS, 2 mM glutamine, 1000 U/mL penicillin, 100 mg/mL Streptomycin. F9 cells were cultured on gelatinized tissue culture plates. All cells were cultured at 37°C in 5% CO2. Viruses were prepared as previously described using pNCA-GFP/mCherry vectors [33] pseudotyped with VSV-G amphotropic envelope glycoprotein. At 48 hr posttransfection, culture supernatants were harvested, filtered through a 0.45 μm filter, concentrated by ultracentrifugation, and resuspended in growth medium.

### Virus copy number determination

The difference in threshold cycle (CT) values (DCT) between Q-PCR assays using two of the primers on the pro-virus, 40NT and PBS, was used to monitor proviral DNA copy number. The Gapdh primer set for the genomic DNA was used to normalize the amount of genomic DNA. A one copy number standard was established by infecting NIH 3 T3 cells at a very low multiplicity of infection (MOI) with the MLV-GFP vector and sorting the GFP(+) cell population (10% of the total), ensuring that a single copy of GFP virus was present [25]. The ratio of GFP to genomic DNA (Gapdh) in this sample is normalized to 1 (one copy of provirus per cell genome), and this sample is taken as the calibrator. The differences in DCTs (DDCT) for the samples of interest and the calibrator are used to estimate the relative quantity (RQ) of provirus by using the formula RQ = 2 ^-DDCT^. The copy number values given were obtained by averaging results from three PCR reactions. Uninfected NIH 3 T3 cells were used as negative control. In the flow analysis results presented, all numbers were normalized to the infection efficiency as seen by the % of NIH3T3 expressing cells. The numbers are given as GFP-positive F9 cells/GFP-positive NIH3T3 cells x100; this is to correct for variation in multiplicity with different viruses.

### Flow cytometry and sorting

GFP-positive cells were isolated on a cell sorter (FACSAria Cell Sorter; BD Biosciences). Data were acquired on an automated cell analyzer (LSR II; BD Biosciencs) and analyzed with FlowJo software (Treestar).

### Bisulfite analysis of methylation

Bisulfite conversion of genomic DNA was carried out using the Zymo EZ DNA Methylation-Gold™ Kit. PCR primers were designed using Methyl Primer Express software version 1.0 (https://www2.appliedbiosystems.com). See Additional file 4: Table S1.

### Chromatin immunoprecipitation (ChIP)

10^7^ cells were crosslinked with 1% formaldehyde at room temperature for 10 min. Chromatin was extracted and then sonicated to an average size of 300–1,000 bp. Immunoprecipitation was carried out by using Magna ChIP™ kit as recommended by the manufacturer (Millipore) and then purified using QIAquick PCR purification kit (Qiagen). Antibodies used (about 5 μg per 10–30 μg of DNA) were: Anti YY1 (H-414, Santa Cruz), Anti Trim28 (Anti tif1b- MAB3662, Millipore), Anti-trimethyl-Histone H3 (Lys9) (07–442, Millipore), Anti-trimethyl-Histone H3 (Lys27) (07–449, Millipore) and Anti-trimethyl-Histone H3 (Lys4) (07–473, Millipore). IP with IgG antibody (sc-2027, Santa Cruz) resulted in enrichment level < 1. Amplification was carried out by real-time PCR, and the bound/input values were then normalized by setting the negative control gene results to 1. Multiple assays of the same sample or the same gene sequence were analyzed in separate immunoprecipitations. All immunoprecipitations were repeated at least 3 times. Primer sequences used for qPCR are listed in Additional file 4: Table S1. In Figure 6D,E bound/input ratios were normalized to the U5-PBS primers value of the same sample and results are presented relative to the value in the negative sorted cells fraction.

## Competing interests

The authors declare that they have no competing interests.

## Authors’ contributions

SS designed the experiments, carried out the work and drafted the manuscript. EM provided advice and help with interpretation of the data and reviewed the findings. SPG designed the program, supervised the work and drafted and edited the manuscript. All authors read and approved the final manuscript.

## Supplementary Material

Additional file 1: Figure S1 Proviral DNA copy number. (A) After infection of F9 cells, the initial unsorted and various sorted populations were analyzed for infection efficiency by qPCR. Results are shown in arbitrary units after normalization to Ctrl = cells with single viral copies. (B and C) NIH3T3 cells were infected with 1/10 of the concentration that was used to infect the F9 cells in panel A, with (B) PBSpro – GFP and mCherry viruses or with (C) PBSgln – GFP and mCherry viruses, and followed for expression of the markers by flow analysis. On the right is an example of the flow analysis dot plot recorded 27 days after infection (white) and in comparison to an uninfected control (black). Click here for file

Additional file 2: Figure S2 Expression of reporter genes in subclones over time after sorting for double-positive populations. Panels A-E: Double-positive cells sorted after infection with PBSpro virus were subcloned and analyzed by flow cytometry for 40 days after sorting. Results show variability in expression levels in five individual clones that is possibly affected by the various integration sites of the proviruses in the clones. Click here for file

Additional file 3: Figure S3 CpG methylation in sorted populations is similar whether selected for single or medium double-positive expression. Clones from the indicated populations (A-C) were scored for %mGC at either the GFP locus (left) or mCherry locus (right). Values are average of 10 clones. Results for examples from each population are shown. Click here for file

Additional file 4: Table S1 Primer and Probe List. Click here for file
