# Supplementary figures and images for: Asynchronous transcriptional silencing of individual retroviral genomes in embryonic cells
Source: Retrovirology. 2014 Apr 17;11:31. doi: 10.1186/1742-4690-11-31 (PMC4021621; doi:10.1186/1742-4690-11-31)

A

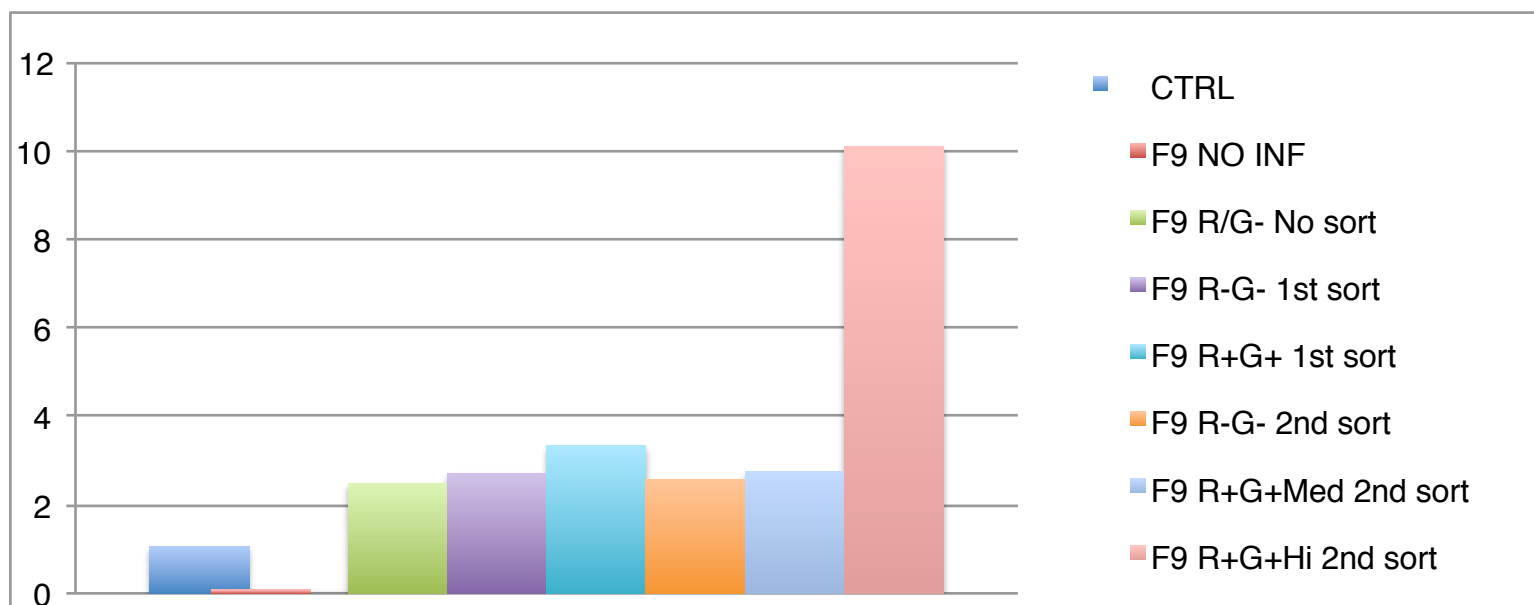

B

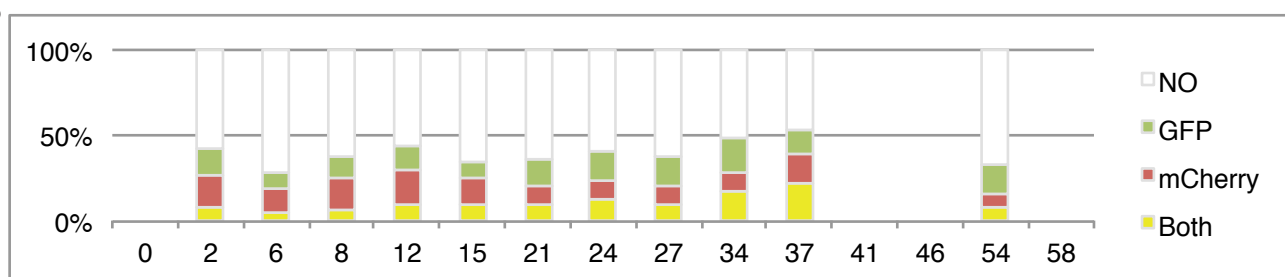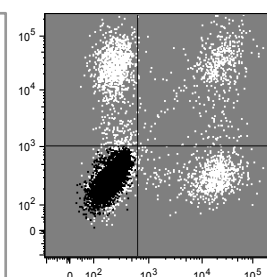

C

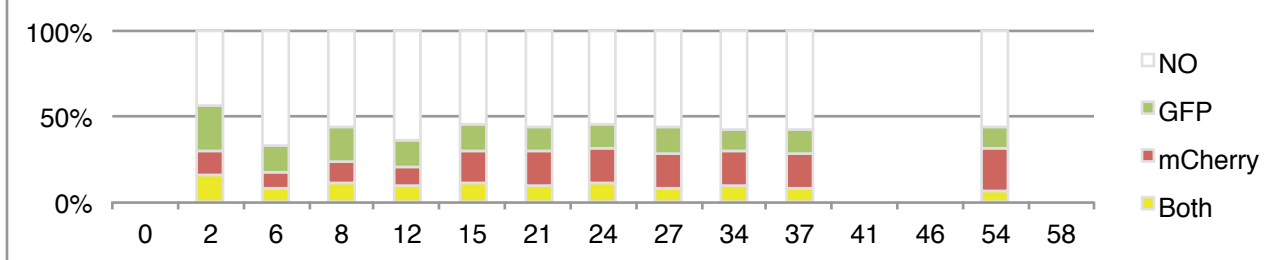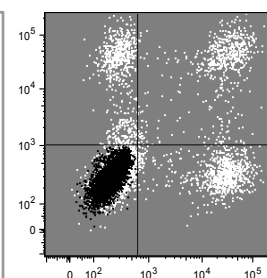

Supplement: Additional file 1: Figure S1 — Proviral DNA copy number. (A) After infection of F9 cells, the initial unsorted and various sorted populations were analyzed for infection efficiency by qPCR. Results are shown in arbitrary units after normalization to Ctrl = cells with single viral copies. (B and C) NIH3T3 cells were infected with 1/10 of the concentration that was used to infect the F9 cells in panel A, with (B) PBSpro – GFP and mCherry viruses or with (C) PBSgln – GFP and mCherry viruses, and followed for expression of the markers by flow analysis. On the right is an example of the flow analysis dot plot recorded 27 days after infection (white) and in comparison to an uninfected control (black). [file 1742-4690-11-31-S1.pdf]

A

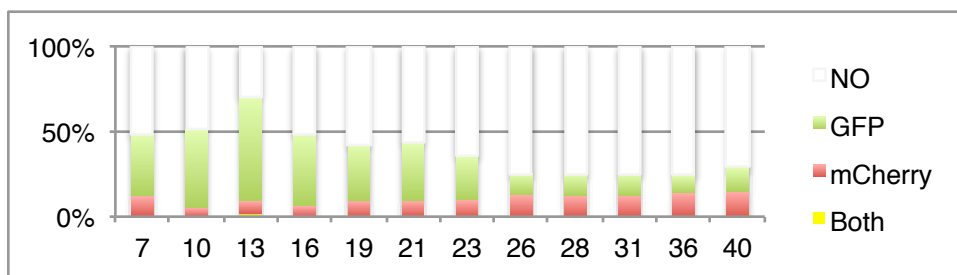

B

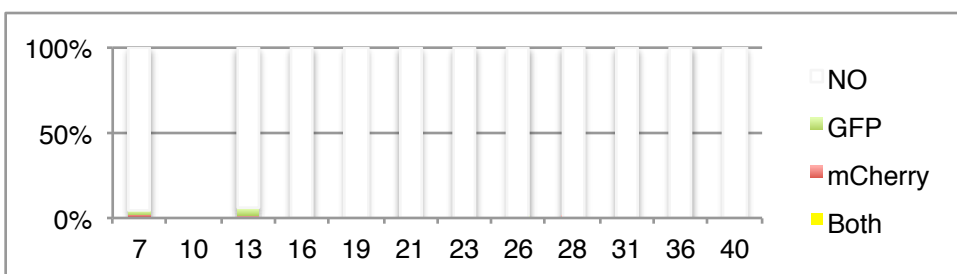

C

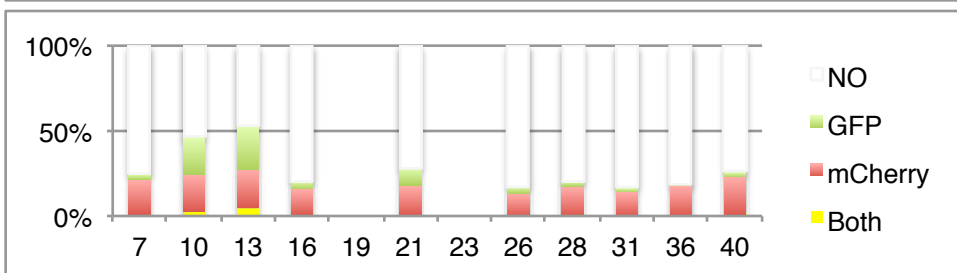

D

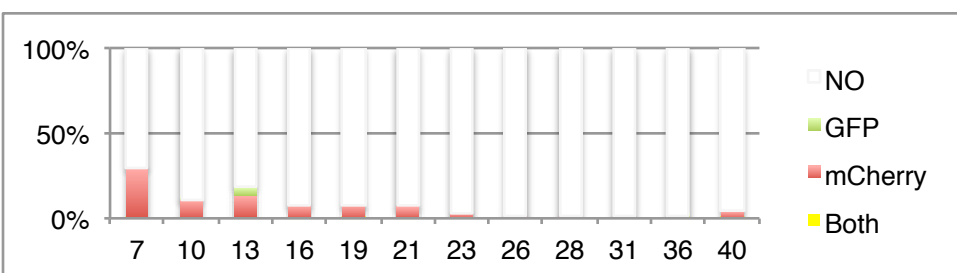

E

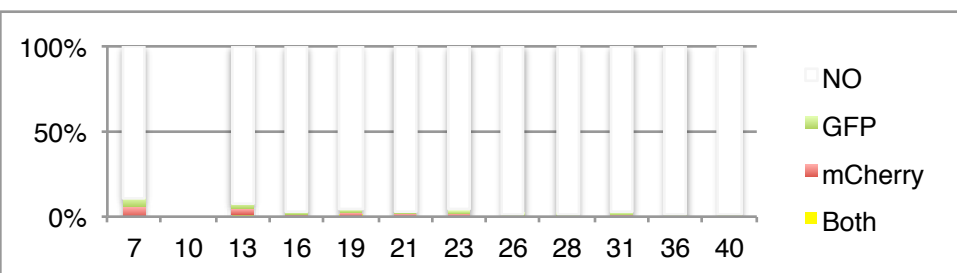

Supplement: Additional file 2: Figure S2 — Expression of reporter genes in subclones over time after sorting for double-positive populations. Panels A-E: Double-positive cells sorted after infection with PBSpro virus were subcloned and analyzed by flow cytometry for 40 days after sorting. Results show variability in expression levels in five individual clones that is possibly affected by the various integration sites of the proviruses in the clones. [file 1742-4690-11-31-S2.pdf]

## A F9 R-G+ - GFP (31% mC)

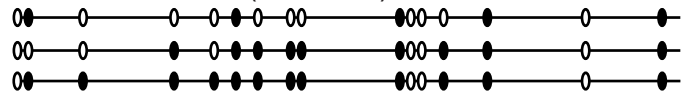

## F9 R-G+ - mCherry (54% mC)

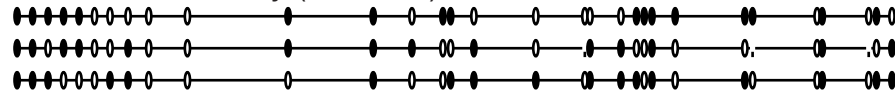

## B F9 R+G- - GFP (64% mC)

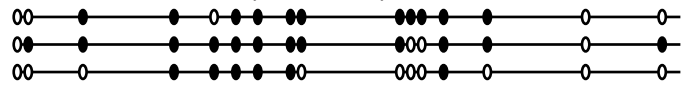

## F9 R+G- - mCherry (40% mC)

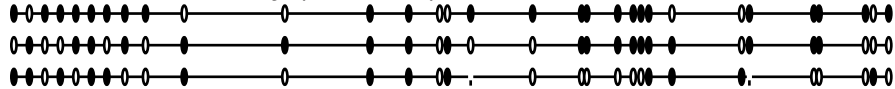

## C F9 R+G+ Med - GFP (46% mC)

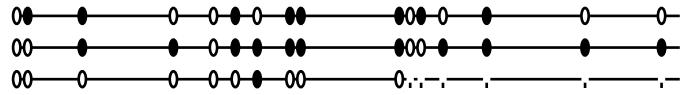

## F9 R+G+ Med - mCherry (52% mC)

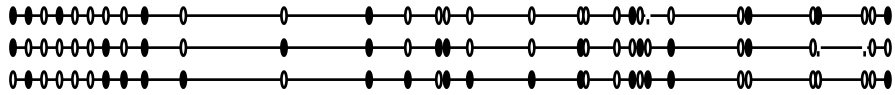

Supplement: Additional file 3: Figure S3 — CpG methylation in sorted populations is similar whether selected for single or medium double-positive expression. Clones from the indicated populations (A-C) were scored for %mGC at either the GFP locus (left) or mCherry locus (right). Values are average of 10 clones. Results for examples from each population are shown. [file 1742-4690-11-31-S3.pdf]
