# Supplementary material for: Asynchronous transcriptional silencing of individual retroviral genomes in embryonic cells
Source: Retrovirology. 2014 Apr 17;11:31. doi: 10.1186/1742-4690-11-31 (PMC4021621; doi:10.1186/1742-4690-11-31)
Supplement: Additional file 4: Table S1 — Primer and Probe List. [file 1742-4690-11-31-S4.pdf]

Additional table 1. Primer list

Taqman primers and probes (used for ChIP and Copy number determination)	taq-40nt1-F	AAT GAA AGA CCC CAC CTG TAG GT
	taq-40nt94-R	AAA TGG CGT TAC TTA AGC TTG GA
	taq-40nt40-Probe	/56-FAM/TCC TCC ACA CAC CAT CAC TCA CTC TTT CTC /36-TAMSp/
	taq-PBS589-F	AGG GTC TCC TCT GAG TGA TTG ACT
	taq-PBS731-R	TCG GAC AGA CAC AGA TAA GTT GCT
	taq-PBS653-Probe	/56-FAM/ATC GGG AGA CCC CTG CCC AGG /36-TAMSp/
ChIP primers (qPCR, Syber Green)	U5-PBS-F	TGG CCA GCA ACT TAT CTG TG
	U5-PBS-R	CAG GCG CAT AAA ATC AGT CA
	mCherry2-R	GCC GTC CTC GAA GTT CAT CA
	mCherry2-L	CCG ACA TCC CCG ACT ACT TG
	GFP2-L	AGC TGA AGG GCA TCG ACT T
	GFP2-R	GAT GCC GTT CTT CTG CTT GT
	MLV PBS CHIP, F	GTA AAA ACT CCA CAC TCG GC
	MLV PBS CHIP, R	ACG ATT CGG ATG CAA ACA GC
	GLN_631 F	CGT AAG GAC CCT AGT GGC TG
	GLN_784 R	GCA CTC ACT CTT CTT CAC TCT G
	IAP PBS CHIP, F	CGT GAG AAC GCG TCG AAT AA
	IAP PBS CHIP, R	TTC TGG TTC TGG AAT GAG GG
	IAP Pol F	CTT GCC CTT AAA GGT CTA AAA GCA
	IAP Pol R	GCG GTA TAA GGT ACA ATT AAA AGA TAT GG
	L-mChip-Polrmt	AGA CAC CTG CTG CCC TAT GT
	R-mChip-Polrmt	GCT CCA TCC CAG TGC TTT AC
	L-mChip-mest	CGT GGT CCA AGG GAT ATG TT
	R-mChip-mest	TGA AGA AAG CCT TCC CAT GT
	mES_Bmp2_Fd	CCG ATC ACC TCT CTT CCT CA
	mES_Bmp2_Rev	CTG GGC TTC TGT TGC TTT TC
	L-mChip-Peg13	AGC CTC TGT GCT AGC GTC TC
	R-mChip-Peg13	GGA TAC CTT CGA GCG TTG AG
	Bmp2_Fd	CCG ATC ACC TCT CTT CCT CA
	Bmp2_Rev	CTG GGC TTC TGT TGC TTT TC
	L-mChIP-Peg3	GGA GGC GGT GTC TGA AGT AA
	R-mChIP-Peg3	CAG AGC TCC CTG CTC ATT CT
	L-mChIP-5'Fkbp6	CAT GCT CGC TGC GTC TAT C
	R-mChIP-5'Fkbp6	ATC TTG CCG CAC AAC TGT CT
	L-Gapdh	ACC TTT AGC CTT GCC CTT T
	R-Gapdh	ACA TCA CCC CCA TCA CTC AT

	L-m-Aprt	GGG ATA TCT CGC CCC TCT T
	R-m-Aprt	CAC TCG CCT GCG ATG TAG T
RT-qPCR primers	CYCAexon3-L	GAG CTC TGA GCA CTG GAG AGA
	CYCAexon3/4-R	CCA CCC TGG CAC ATG AAT
	UBC-L exon 1	CAG CCG TAT ATC TTC CCA GAC T
	UBC-R exon2	CTC AGA GGG ATG CCA GTA ATC TA
	GAPDH-L	CAG CCG TAT ATC TTC CCA GAC T
	GAPDH-R	CTC AGA GGG ATG CCA GTA ATC TA
	Oct4-exon1/2-L	AAC CAA CTC CCG AGG AGT CCC A
	Oct4-exon2-R	TCT TCT GCT TCA GCA GCT TGG CA
	40nt-L	CTC CTC CAC ACA CCA TCA CT
	out40nt-R	CTG TTC CTG ACC TTG ATC TGA A
	mCherry2-R	GCC GTC CTC GAA GTT CAT CA
	mCherry2-L	CCG ACA TCC CCG ACT ACT TG
	GFP-L	CTC GTG ACC ACC TTG ACC TA
	GFP-R	GAA GAA GTC GTG CTG CTT CA
Bisulfite nested primers	bis40nt(88)L	GTT ATT TTG TAA GGT ATG GAA AAA
	bis40nt(727)R	ACA AAC ACA AAT AAA TTA CTA ACC A
	bis40nt(382)L	GAA ATG ATT TTG TGT TTT ATT TGA A
	bis40nt(715)R	AAA TTA CTA ACC AAC TTA CCT CCC
	bisGFP3-F	GAG TTG AAG GGT ATY GAT TTT AA
	bisGFP3-R	CCC CCA TAC TCR AAA CAT A
	bisGFP1-F	TGG GGT ATA AGT TGG AGT ATA A
	bisGFP1-R	TTC TCR TTA AAA TCT TTA CTC AA
	bisMcherry1 F	TTT AAG GTG TAT ATG GAG GGT T
	bisMcherry1 R	CAA CCC ATA ATC TTC TTC TAC A
	bisOct4extF	AAT TTT ATT TTT TAG TTT TGA TTT TTG G
	bisOct4extR	TAA ACA AAA ACT AAA AAA TAA CCC CAA AAA T
	bisOct4intF	TGG GTG GGT GGA GGA GTA GAG TTG TGG GGG
	bisOct4intR	CTC TCC CAA AAA ATA ACT AAA TAA ACT ATA AAA
